# Implementation of Sound Direction Detection and Mixed Source Separation in Embedded Systems

**DOI:** 10.3390/s24134351

**Published:** 2024-07-04

**Authors:** Jian-Hong Wang, Phuong Thi Le, Weng-Sheng Bee, Wenny Ramadha Putri, Ming-Hsiang Su, Kuo-Chen Li, Shih-Lun Chen, Ji-Long He, Tuan Pham, Yung-Hui Li, Jia-Ching Wang

**Affiliations:** 1 School of Computer Science and Technology, Shandong University of Technology, Zibo 255000, China; jhwang@sdut.edu.cn (J.-H.W.); 23505020692@stumail.sdut.edu.cn (J.-L.H.); 2 Department of Computer Science and Information Engineering, Fu Jen Catholic University, New Taipei City 242062, Taiwan; ptle@csie.fju.edu.tw; 3 Department of Computer Science and Information Engineering, National Central University, Taoyuan City 320314, Taiwan; wengshengbee2@hotmail.com (W.-S.B.); wenny@g.ncu.edu.tw (W.R.P.); jcw@csie.ncu.edu.tw (J.-C.W.); 4 Department of Data Science, Soochow University, Taipei City 10048, Taiwan; 5 Department of Information Management, Chung Yuan Christian University, Taoyuan City 320317, Taiwan; 6 Department of Electronic Engineering, Chung Yuan Christian University, Taoyuan City 320314, Taiwan; chrischen@cycu.edu.tw; 7 Faculty of Digital Technology, University of Technology and Education—University of Đà Nẵng, Danang 550000, Vietnam; ptuan@ute.udn.vn; 8 AI Research Center, Hon Hai Research Institute, New Taipei City 207236, Taiwan; yunghui.li@foxconn.com

**Keywords:** embedded systems, position detection, hybrid sound source separation, signal-to-interference ratio (SIR), speech recognition

## Abstract

In recent years, embedded system technologies and products for sensor networks and wearable devices used for monitoring people’s activities and health have become the focus of the global IT industry. In order to enhance the speech recognition capabilities of wearable devices, this article discusses the implementation of audio positioning and enhancement in embedded systems using embedded algorithms for direction detection and mixed source separation. The two algorithms are implemented using different embedded systems: direction detection developed using TI TMS320C6713 DSK and mixed source separation developed using Raspberry Pi 2. For mixed source separation, in the first experiment, the average signal-to-interference ratio (SIR) at 1 m and 2 m distances was 16.72 and 15.76, respectively. In the second experiment, when evaluated using speech recognition, the algorithm improved speech recognition accuracy to 95%.

## 1. Introduction

In recent years, embedded system technology and products have become the focus of the global IT industry. As people pursue a more convenient and comfortable lifestyle, the information industry and smart homes are booming. Embedded systems are increasingly integrated into our daily lives in various forms, such as sensor networks and wearable devices for monitoring people’s activities and health. Although many people still do not fully understand what embedded systems are, they are closely related to our daily lives and have already permeated various fields, such as home applications [1,2,3,4,5,6,7], wireless communications [4,8], network applications [9,10], medical devices [4,11,12], consumer electronics, etc. Embedded systems encompass many applications, including smart homes, gaming consoles, electronic stethoscopes, automated teller machines (ATMs), and car-mounted Global Positioning Systems (GPSs).

This paper discusses the implementation of audio localization and enhancement in embedded systems, focusing on embedded algorithms for direction detection and mixed sound source separation. These two algorithms are implemented using different embedded systems: the TI TMS320C6713 DSK [13,14,15,16,17,18,19,20] for direction detection development and the Raspberry Pi 2 [21,22,23,24,25] for mixed sound source separation. The objective is to develop audio localization and noise reduction techniques applicable to intelligent living to bring convenience and comfort to users’ lives.

Direction detection entails capturing audio from a microphone array and determining the direction of the sound source through a specialized algorithm. Azimuth detection is utilized for audio tracking, with the TDE method [26] employed for direction detection. By utilizing Cross-Power Spectral Density (XPSD) based on the Generalized Cross-Correlation with Phase Transform (GCC-PHAT) estimate and detecting the peak of cross-correlation, we can accurately identify the azimuthal relationship between the sound signal and the microphone array.

The current research on direction detection, domestically and internationally, can be divided into two categories. The first category utilizes beamforming or subspace theory in conjunction with microphone arrays to determine the angle of the sound source. The most widely used method in this category is Multiple Signal Classification (MUSIC) [27]. The second category employs Time Delay of Arrival (TDOA) to estimate the angle of the sound source based on the time delay between the arrival of the sound at different microphones [28,29]. Among these, the Generalized Cross-Correlation (GCC) method proposed by Kanpp and Carter [26] is considered one of the most common TDOA methods. The first research category requires prior measurement of the impulse frequency response corresponding to each microphone in the array, resulting in significant computational complexity. Considering the real-time nature of the system, this paper will adopt the Generalized Cross-Correlation PHAT [26,30] method.

Mixed sound source separation involves extracting multiple individual source signals from the mixed signal captured by the microphone. Since the inception of this problem, it has garnered significant attention from researchers. We aim to extract the desired sounds embedded within the observed signal using mixed sound source separation techniques. Blind source separation of mixed signals can be classified in two ways based on the type of mixing model: the instantaneous mixing model [31,32] and the convolution mixing model [33,34,35,36]. Our method is as follows [37]. First, the Fourier transform transfers the received mixed signal to the frequency domain, and then features are extracted and input into K-Means to cluster the signal, where the k value is set to 2, as there are two mixed sound sources. Next, the mixed signal is subjected to Binary Masking to reconstruct the source in the frequency domain. The signal is finally converted back to the time domain using the inverse Fourier transform. This paper primarily explores the convolution mixing model as it finds application in real-world environments.

Due to the limited processing power and memory capacity of embedded system processors compared to PCs, efficient utilization of memory, computational resources, and program storage space become critical in the embedded system environment. Therefore, we need to further optimize the computational load in the algorithm and streamline the code to ensure smooth execution in embedded systems. Code Composer Studio (CCS) is an integrated development environment developed by TI. It provides an optimized compiler that compiles program code into efficient executable programs. CCS also provides a real-time operating system, DSP/BIOS, which can provide simple and effective management of programs. This study will utilize CCS to optimize the computational load and ensure smooth execution in embedded systems. To enhance the speech recognition capabilities of wearable devices, the contribution of this article is to realize audio positioning and enhancement in embedded systems, use embedded algorithms to perform direction detection and mixed source separation, and ultimately increase the speech recognition accuracy, further improving the practicality of wearable devices.

## 2. Embedded System Design for Direction Detection

### 2.1. Algorithm Flow and Overview

**Figure 1:**
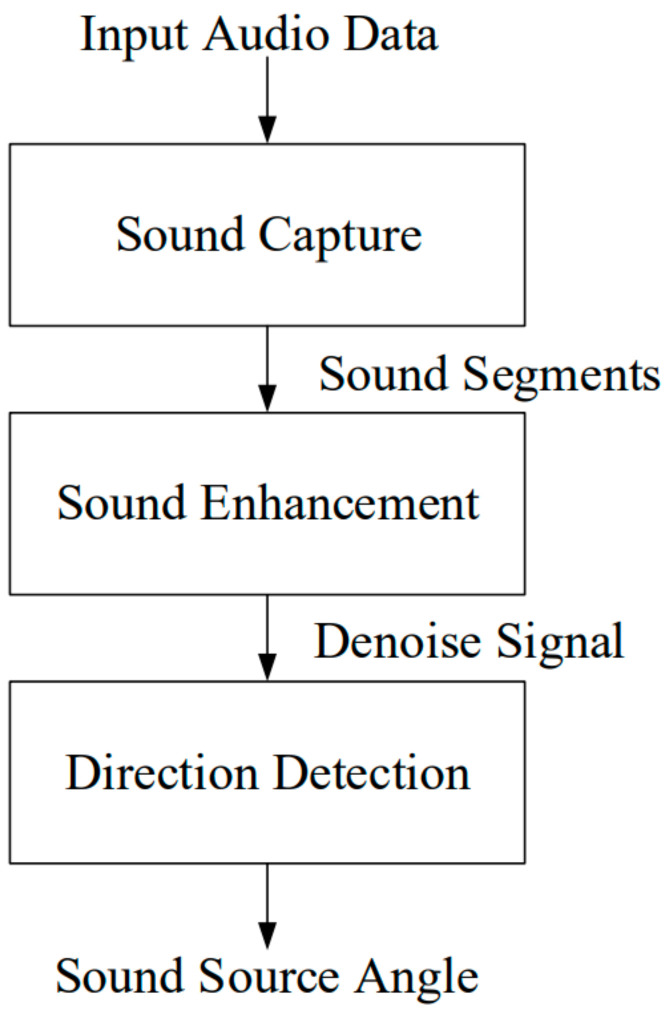
Architecture diagram of the embedded system for direction detection.

Firstly, we conduct voice activity detection (VAD) preprocessing on the received audio signal to identify segments containing speech [38]. Subsequently, we utilize the spectral subtraction method [39,40] to remove noise from the audio. Finally, the denoised audio is forwarded to the DOA (Direction of Arrival) recognizer for direction detection [26,30]. Figure 1 illustrates the architecture of the embedded system proposed in this paper for direction detection.

#### 2.1.1. Voice Activity Detection (VAD)

We use conventional energy-based Voice Activity Detection (VAD) [38] to extract sound events. Let $x_{t}\left(n\right)$ represent the received audio, where t indicates the audio frame and n ranges from 1 to N samples. ${Μ}_{t}$ denotes the average value of audio $x_{t}$. $E_{t}$ is determined by a threshold value, T, resulting in either A = 1 for sound events or A = 0 for non-sound events. Since different microphones may have different threshold values, it is necessary to conduct testing to determine the appropriate threshold value.

(1) $$E_{t}=\sum_{n=1}^{N}{{(x}_{t}\left(n\right)-\mu_{t})}^{2}$$

(2) $$A=\left\{\begin{array}{c} 1,\;\;\;\;\;\;E_{t}>T\;\;\;\;\;\;\;\\ 0,\;otherwise \end{array}\right.$$

#### 2.1.2. Sound Enhancement—Spectral Subtraction

Sound enhancement is achieved using the spectral subtraction method [39,40]. The benefit of the spectral subtraction method over some machine learning-based sound enhancement methods [41,42,43] is its lower computational complexity. This method involves subtracting the averaged noise spectrum from the spectrum of the noisy signal to eliminate environmental noise. The averaged noise spectrum is obtained from the signals received during non-sound events.

If the noise, n(k), of one audio frame is added to the original signal, $s\left(k\right)$, of the same audio frame, resulting in a noisy signal $s\left(k\right)$ for that frame, we have the following equation:

(3) $$y\left(k\right)=s\left(k\right)+n\left(k\right)$$

After performing the Fourier transform, we obtain the following:

(4) $$Y\left(e^{j\omega }\right)=S\left(e^{j\omega }\right)+N\left(e^{j\omega }\right)$$

The general formula for the spectral subtraction method is as follows:

(5) $${\left|S_{S\left(e^{j\omega }\right)}\right|}^{2}={\left|Y\left(e^{j\omega }\right)\right|}^{2}-\alpha {\left|\mu \left(e^{j\omega }\right)\right|}^{2},\;\mathrm{if}\;{\left|Y\left(e^{j\omega }\right)\right|}^{2}>\alpha {\left|\mu \left(e^{j\omega }\right)\right|}^{2}\beta {\left|Y\left(e^{j\omega }\right)\right|}^{2},\;{\left|\mu \left(e^{j\omega }\right)\right|}^{2}=E\left\{{\left|N\left(e^{j\omega }\right)\right|}^{2}\right\}$$

where $\mu \left(e^{j\omega }\right)$ represents the average noise spectrum, α lies between 0 and 1, and β is either 0 or a minimum.

After subtracting the spectral energy, we obtain the denoised signal spectrum $\hat{S}\left(e^{j\omega }\right)$. $\theta_{Y}\left(e^{j\omega }\right)$ represents the phase of $Y\left(e^{j\omega }\right)$.

(6) $$\hat{S}\left(e^{j\omega }\right)=\left|S_{S\left(e^{j\omega }\right)}\right|e^{j\theta_{Y}\left(e^{j\omega }\right)}$$

Alternatively, by obtaining the ratio $H\left(e^{j\omega }\right)$ of the energy-subtracted spectrum ${\left|S_{S\left(e^{j\omega }\right)}\right|}^{2}$ to the spectrum of the noisy signal ${\left|Y\left(e^{j\omega }\right)\right|}^{2}$, we multiply it with $Y\left(e^{j\omega }\right)$ to obtain the denoised signal spectrum $\hat{S}\left(e^{j\omega }\right)$.

(7) $$\hat{S}\left(e^{j\omega }\right)=H\left(e^{j\omega }\right)Y\left(e^{j\omega }\right)$$

(8) $$H\left(e^{j\omega }\right)=\frac{{\left|S_{S\left(e^{j\omega }\right)}\right|}^{2}}{{\left|Y\left(e^{j\omega }\right)\right|}^{2}}$$

#### 2.1.3. Direction Detection—TDE-to-DOA Method

We referred to related papers that utilize the GCC-PHAT [26,30] estimation for the XPSD (Cross-Power Spectral Density) and the peak detection of cross-correlation for direction detection using the Time Delay Estimation (TDE) method. In addition, we also refer to the research of Varma et al. [44], which uses cross-correlation-based time delay estimates (TDE) for direction-of-arrival (DOA) estimation of acoustic arrays in less reverberant environments. The TDE method determines the direction of a single sound source, and multiple sound sources cannot be differentiated simultaneously. However, its advantage lies in its simplicity, as it only requires two microphones and has a relatively straightforward hardware architecture, making it suitable for real-time applications.

Firstly, we assume the presence of a sound source in the space. Under ideal conditions, the signals received by the two microphones can be represented as follows:

(9) $$x_{1}\left(t\right)=s_{1}\left(t\right)+n_{1}\left(t\right)$$

(10) $$x_{2}\left(t\right)=\alpha s_{1}\left(t-D\right)+n_{2}\left(t\right)$$

$s_{1}\left(t\right)$ represents the sound source; $x_{1}\left(t\right)$ and $x_{2}\left(t\right)$ represent the signals received by the two microphones. $n_{1}\left(t\right)$ and $n_{2}\left(t\right)$ are the noises present.

We assume $s_{1}\left(t\right)$, $n_{1}\left(t\right)$, and $n_{2}\left(t\right)$ to be wide-sense stationary (WSS) and $s_{1}\left(t\right)$ and $n_{1}\left(t\right)$, as well as $n_{2}\left(t\right)$, to be uncorrelated. Here, D represents the actual delay, and α represents the scale value for changing the magnitude. Furthermore, the changes in D and α are slow, and at this stage, the cross-correlation between the microphones can be expressed as follows:

(11) $$R_{x1,x2}\left(\tau \right)=E\left[x_{1}\left(t\right)x_{2}\left(t-\tau \right)\right]$$

where *E* represents the expectation value, and τ, which maximizes Equation (11), is the time delay between the two microphones. Since the actual observation time is finite, the estimation of cross-correlation can be expressed as follows:

(12) $${\hat{R}}_{x1,x2}\left(\tau \right)=\frac{1}{T-\tau }\int_{\tau }^{T}x_{1}\left(t\right)x_{2}\left(t-\tau \right)dt$$

where *T* represents the observation time interval, and the relationship between cross-correlation and cross-power spectrum can be expressed in the following Fourier representation:

(13) $$R_{x1,x2}\left(\tau \right)=\int_{-\infty }^{\infty }G_{x1,x2}\left(f\right)e^{j2\pi f\tau }df$$

Now, let us consider the actual state of the physical space, where the sound signals received by the microphones undergo spatial transformations. Therefore, the actual cross-power spectrum between the microphones can be represented as follows:

(14) $$G_{y1,y2}\left(f\right)=H_{1}\left(f\right)H_{2}^{*}\left(f\right)G_{x1,x2}\left(f\right)$$

where $H_{1}\left(f\right)$ and $H_{2}\left(f\right)$ represent the spatial transformation functions from the sound source to the first microphone and the second microphone, respectively. Therefore, we define the generalized correlation between the microphones as follows:

(15) $$R_{x1,x2}^{\left(g\right)}\left(\tau \right)=\int_{-\infty }^{\infty }\Psi_{g}\left(f\right)G_{x1,x2}\left(f\right)e^{j2\pi f\tau }df$$

wherein

(16) $$\Psi_{g}\left(f\right)=H_{1}\left(f\right)H_{2}^{*}\left(f\right)$$

In practice, due to the limited observation time, we can only use the estimated ${\hat{G}}_{x1,x2}\left(f\right)$ instead of $G_{x1,x2}\left(f\right)$. Therefore, Equation (16) is rewritten as follows:

(17) $${\hat{R}}_{x1,x2}^{\left(p\right)}\left(\tau \right)=\int_{-\infty }^{\infty }\Psi_{p}\left(f\right){\hat{G}}_{x1,x2}\left(f\right)e^{j2\pi f\tau }df$$

Using Equation (17), we can estimate the time delay between the microphones. The choice of $\psi_{p}\left(f\right)$ also has an impact on the estimation of time delay. In this paper, we employ the PHAT (Phase Transform) method proposed by Carter et al. [30], which can be expressed as follows:

(18) $$\Psi_{p}\left(f\right)=\frac{1}{\left|G_{x1,x2}\left(f\right)\right|}$$

This method works remarkably well when the noise distributions between the two microphones are independent. By employing the aforementioned approach, we can accurately detect the azimuth relationship between our sound signal and the microphones.

### 2.2. Embedded System Hardware Devices

**Figure 2:**
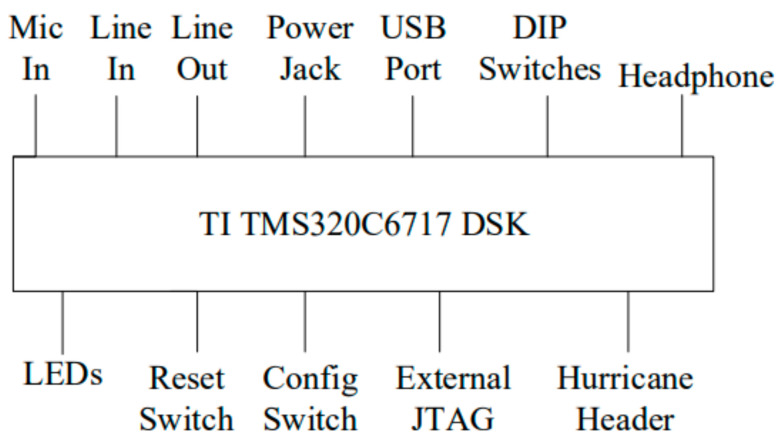
The physical image of the TI TMS320C6713 DSK.

The embedded system for azimuth detection in this study utilizes the TI TMS320C6713 DSK [16,17,18,19,20,21,22,23,37] as the development platform, as shown in Figure 2. In the following sections, we provide a detailed introduction to the specification for the TI TMS320C6713 DSK, which is divided into three parts: peripheral equipment, DSP core, and multi-channel audio input expansion card.

## 3. Design of Embedded System for Separation of Mixed Audio Sources

### 3.1. Algorithm Flow and Introduction

**Figure 3:**
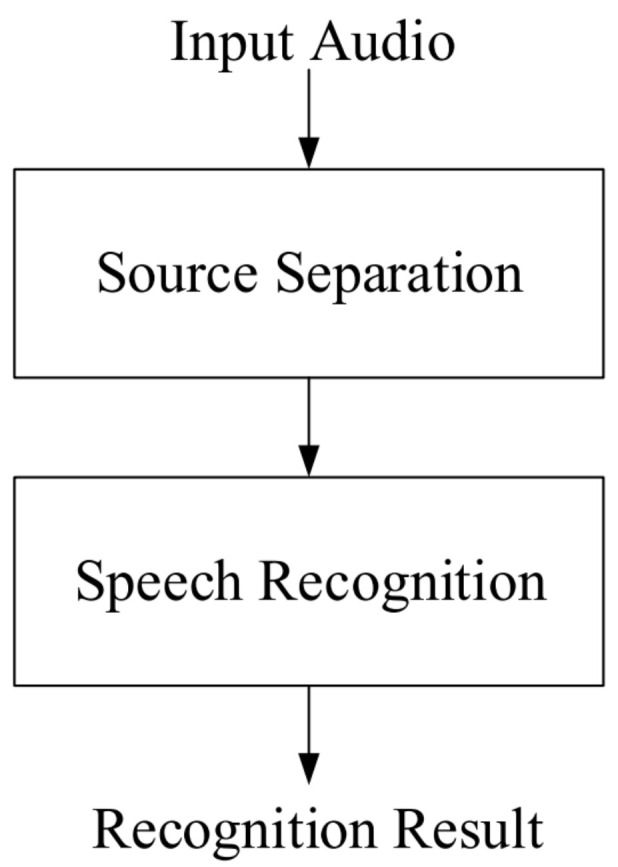
Architecture diagram of a hybrid audio source embedded system.

We begin by applying the blind source separation algorithm [37] to the received audio signal for separating the mixed sources. Then, we upload the separated signals to the Google Speech API for recognition. Figure 3 illustrates the architecture diagram of the embedded system, proposed in this paper, for mixed source separation.

#### Hybrid Audio Source Separation

**Figure 4:**
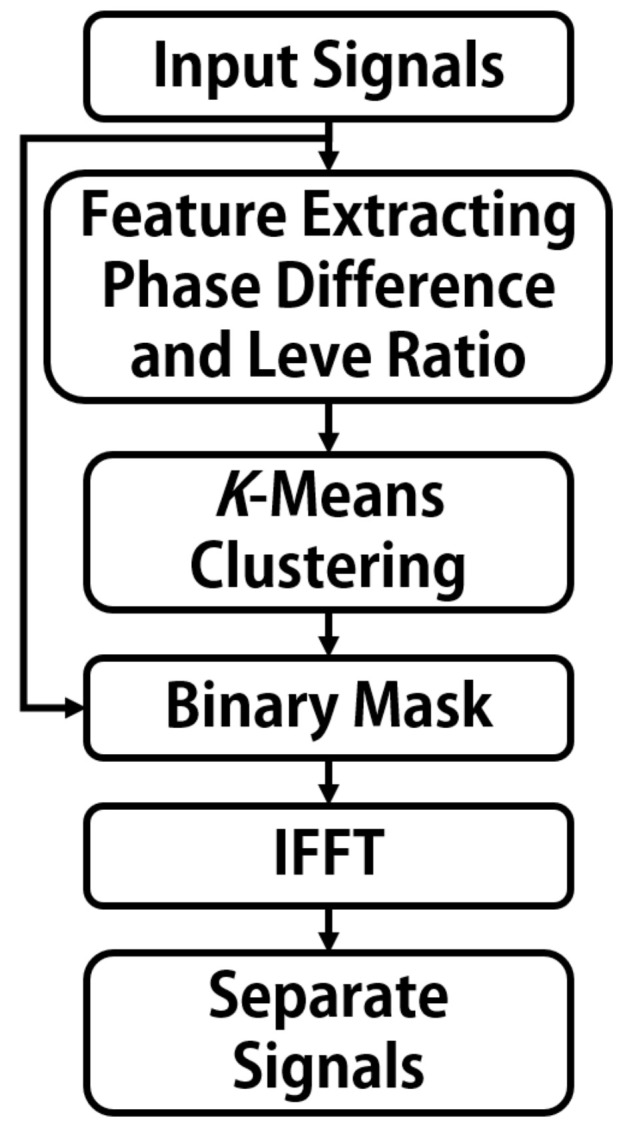
Flowchart of the hybrid audio source separation algorithm.

We set up the system with two receivers (microphones). Initially, the received mixed signal is transformed from the time domain to the frequency domain using Fourier transform to leverage its sparsity for further processing. Then, feature extraction is applied to the transformed signal and input into K-Means for clustering. During the clustering process, corresponding masks are generated, and a Binary Mask is adopted for implementation. Subsequently, Binary Masking is applied to the mixed signal to reconstruct the source signals in the frequency domain. Finally, the signals are transformed back to the time domain using the inverse Fourier transform. Figure 4 illustrates the flowchart of the hybrid audio source separation algorithm [37].

### 3.2. Embedded System Hardware Devices

**Figure 5:**
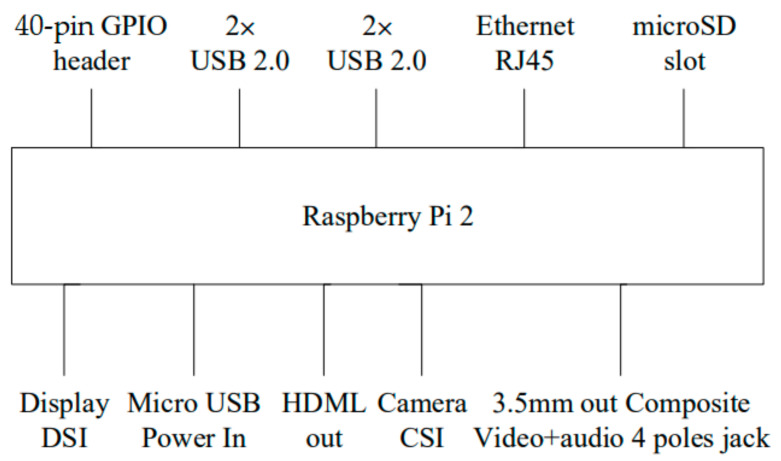
The physical image of the Raspberry Pi 2.

In this paper, Raspberry Pi 2 [21,22,23,24,25] is the development platform for embedding mixed audio sources. The physical diagram is depicted in Figure 5. Here, we will provide a detailed introduction to the specifications for both the Raspberry Pi 2 and the Cirrus Logic Audio Card [45,46] audio module.

#### Cirrus Logic Audio Card Audio Module

**Figure 6:**
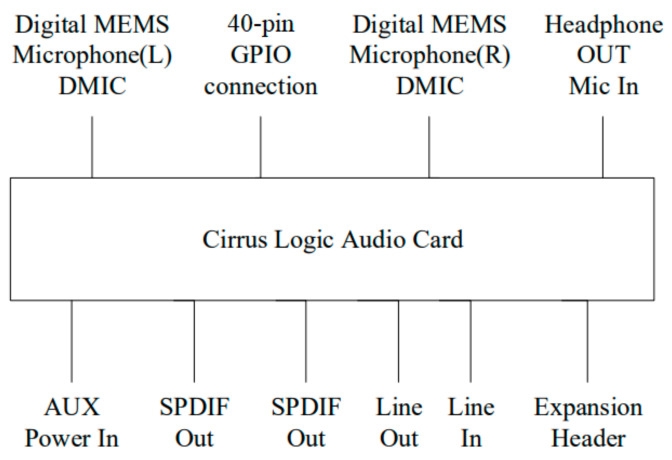
The physical image of the Cirrus Logic Audio Card.

**Figure 7:**
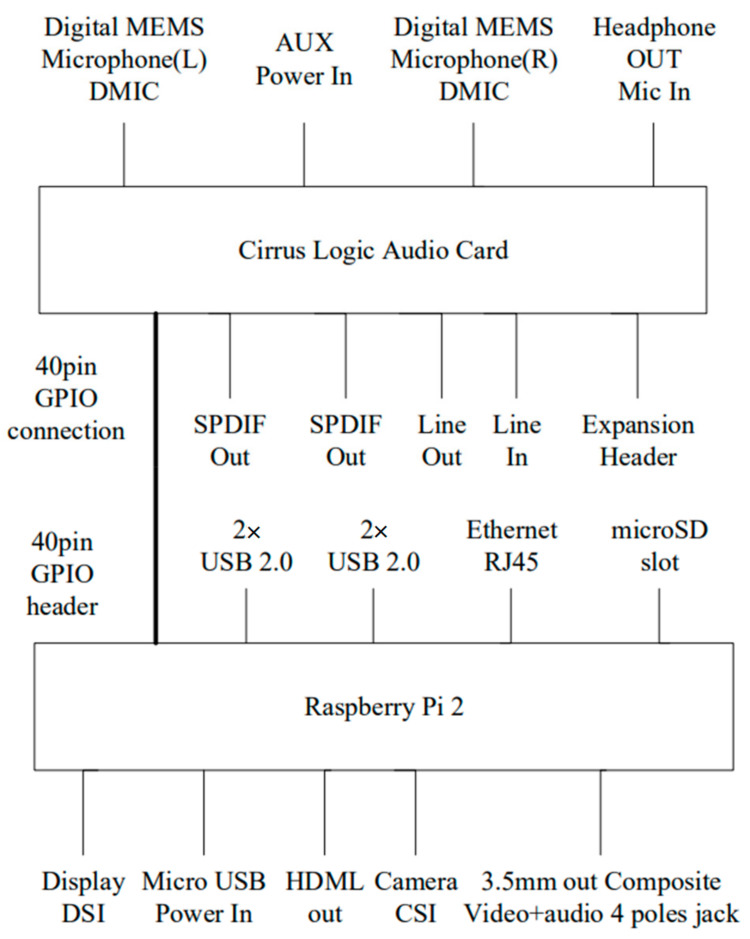
Connection between Raspberry Pi 2 and Cirrus Logic Audio Card.

Figure 6 illustrates the Cirrus Logic Audio Card [45,46], an audio expansion board designed for the Raspberry Pi. Compatible with the Raspberry Pi models A+ and B+, it features a 40-pin GPIO interface that seamlessly connects to the Raspberry Pi’s 40-pin GPIO Header. The card supports high-definition audio (HD Audio) and incorporates two digital micro-electromechanical microphones (DMICs) and D-class power amplifiers for the direct driving of external speakers. The analog signals include line-level input/output and headphone output/headphone microphone input, while the digital signals encompass stereo headphone audio input/output (SPDIF). Moreover, it includes an Expansion Header, enabling connections to devices beyond the Raspberry Pi. Figure 7 depicts the Raspberry Pi 2 connected to the Cirrus Logic Audio Card.

## 4. Experimental Results

### 4.1. Direction Detection of the Embedded System

#### 4.1.1. Experimental Environment Setup

**Figure 8:**
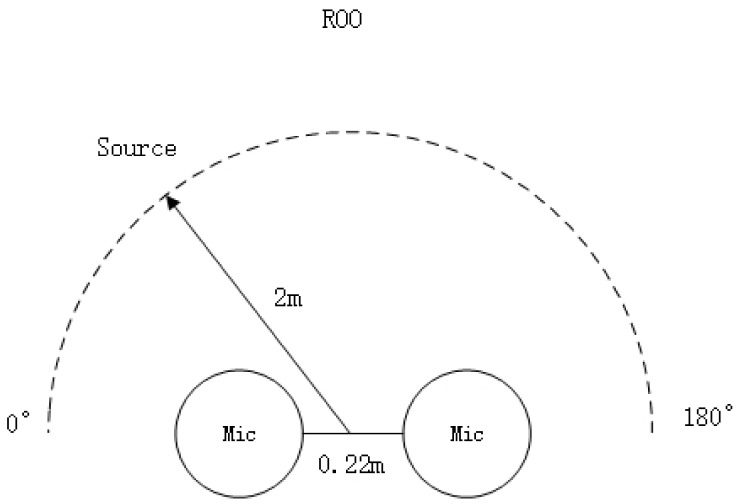
Setup for azimuth detection experiment.

We utilized a classroom measuring 15 m × 8.5 m × 3 m for the experiment. Four omnidirectional microphones were strategically placed within the classroom. The sound source was positioned 2 m away from the center of the microphone array. To assess the azimuth detection capability, we tested 18 angles, ranging from 5° to 175°, with a 10° interval between each test angle (see Table 1 for details). Figure 8 depicts the setup for the azimuth detection experiment.

#### 4.1.2. Experimental Environment Equipment

**Figure 9:**
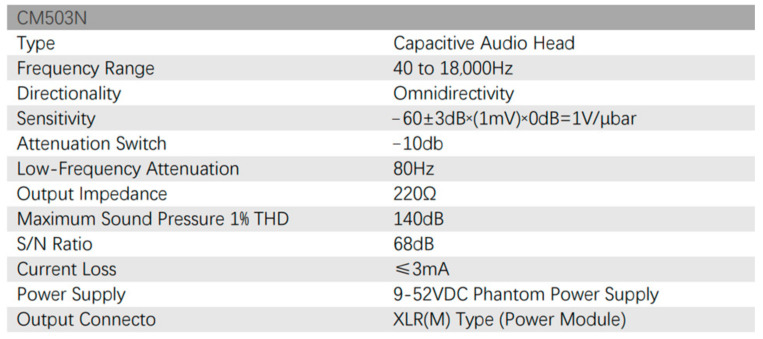
Microphone CM503N.

We utilized the CM503N omnidirectional microphone (depicted in Figure 9). For the equipment setup, the microphone was initially connected to the phantom power supply, and then the phantom power supply was connected to DSK AUDIO 4.

#### 4.1.3. Experimental Results

**Figure 10:**
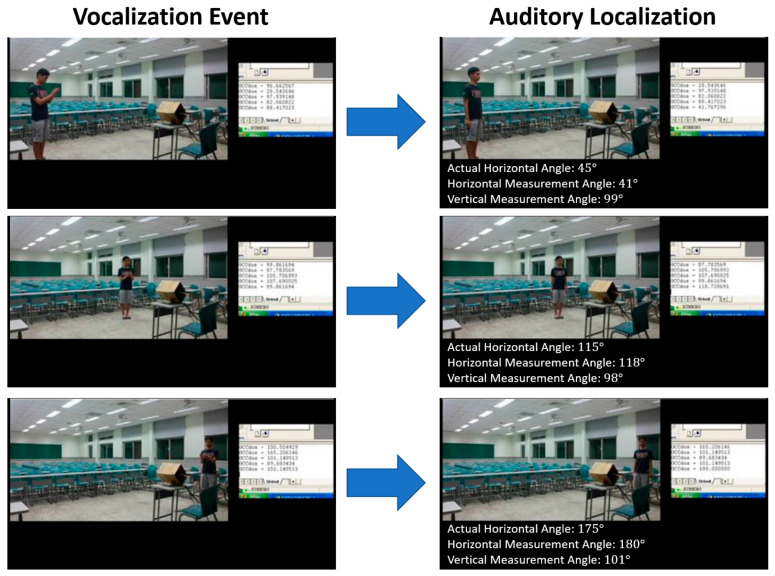
Experimental scenario of direction detection.

The development version of the functionality was completed, and the measured angles yielded satisfactory results, with errors within 10 degrees (refer to Table 2). However, due to the limited memory and processor speed of the development version, achieving real-time measurements is currently not feasible. To address this limitation, a compromise was made by allocating the longest execution time program segments to the smaller internal memory, which offers the fastest execution speed. Meanwhile, the remaining parts were stored in the external memory. This approach ensures a reasonably fast execution speed. Figure 10 depicts the experimental scenario of direction detection.

Based on the current execution results, most of the sound events that surpassed the threshold value during angle measurement achieved an error within 10 degrees. However, there were occasional instances where either no angle measurement was obtained for detected sound events or the measured result was close to 90 degrees. We speculate that the former is attributable to the development board being engaged in other tasks at the time of emitting sound. This resulted in no audio data being captured, thus classifying the signal as silent when determining threshold value passage. As for the latter, we infer that the emitted sound surpassed the threshold value, but it was either too soft and was overshadowed by noise, or the captured sound was too limited, leading to noise being mistakenly identified as a measurable sound event.

### 4.2. Embedded System for Mixed Sound Source Separation

#### 4.2.1. Experimental Environment Setup

**Figure 11:**
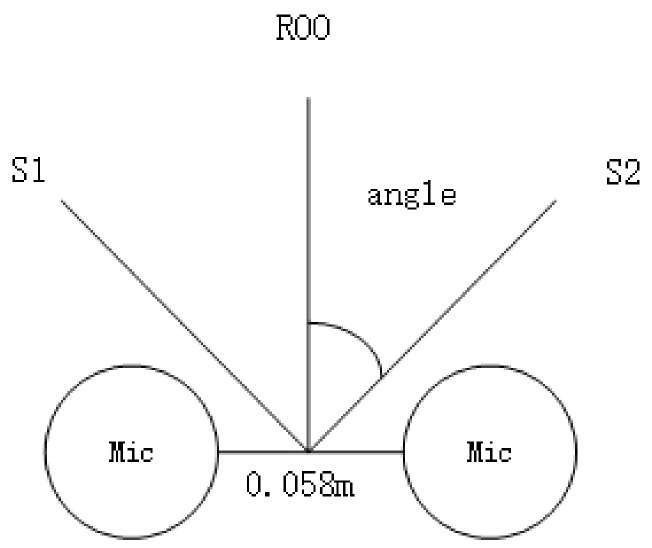
Experimental Environment 1 for mixed sound source separation.

Experimental Setup 1: We utilized a classroom with dimensions of 5.5 m × 4.8 m × 3 m for the experiment. The microphone setup included the two built-in, omnidirectional MEMS microphones from the Cirrus Logic Audio Card, with a distance of 0.058 m between them. The sound sources, denoted as S1 and S2, were positioned at distances of 1 m and 2 m, respectively, from the center of the microphones. S1 was a male speaker, and S2 was a female speaker. Figure 11 illustrates the environment for the mixed sound source separation experiment, and Table 3 provides the setup details for the mixed sound source separation environment.

**Figure 12:**
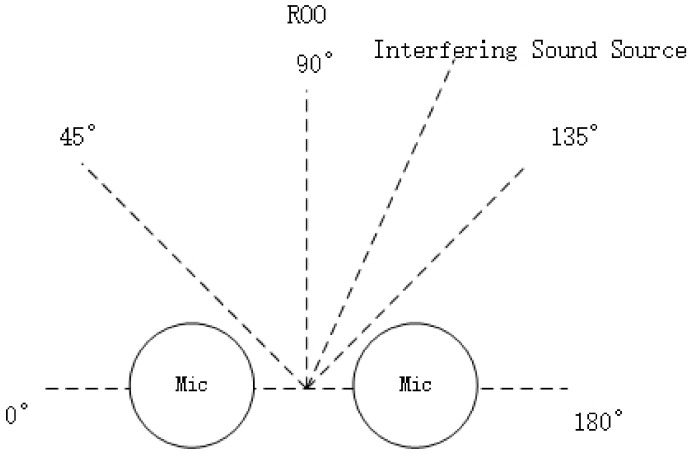
Experimental Environment 2 for mixed sound source separation.

Experimental Setup 2: We utilized a classroom with dimensions of 5.5 m × 4.8 m × 3 m for the experiment. The microphone setup included the two built-in, omnidirectional MEMS microphones from the Cirrus Logic Audio Card, with a distance of 0.058 m between them. The speaker was positioned at a distance of 0.2 m from the center of the microphones, while the interfering sound source (a news broadcast) was positioned at a distance of 1 m from the center of the microphones. Figure 12 illustrates the environment for the mixed sound source separation experiment, and Table 4 provides the setup details for the mixed sound source separation environment.

#### 4.2.2. Experimental Environment Equipment

**Figure 13:**
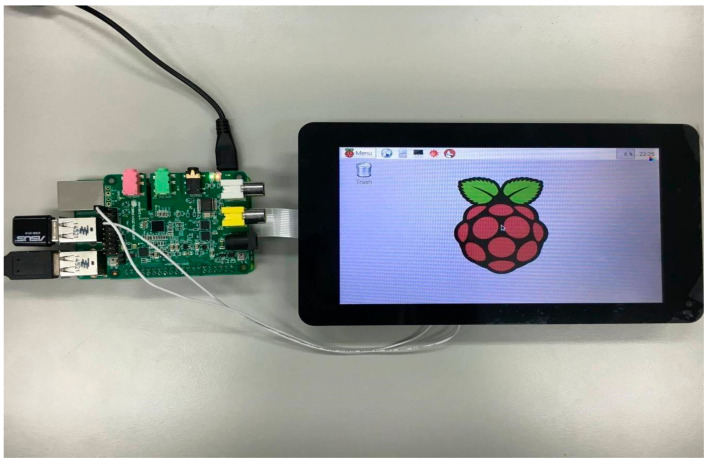
Raspberry Pi 2, Cirrus Logic Audio Card, and 7-inch touch screen.

We utilized a Raspberry Pi 7-inch touch screen (Figure 13) for display purposes, which could be connected to the Raspberry Pi 2 using DSI as the output. Additionally, we utilized the USB-N10 wireless network card for internet access and Google speech recognition.

#### 4.2.3. Experimental Results

For Experimental Setup 1, the signal-to-interference ratio (SIR) served as the performance evaluation metric. The formula for the SIR is as follows:

(19) $$SIR=10{log}_{10}\frac{\left\|y_{qtarget}\right\|}{{\left\|e_{qinterf\;}\right\|}^{2}}$$

where $y_{qtarget}$ represents the components of the source signal in the separated signal, and $e_{qinterf}$ refers to the remaining interference components in the separated signal. Table 5 and Table 6 present the SIR obtained at different distances.

**Figure 14:**
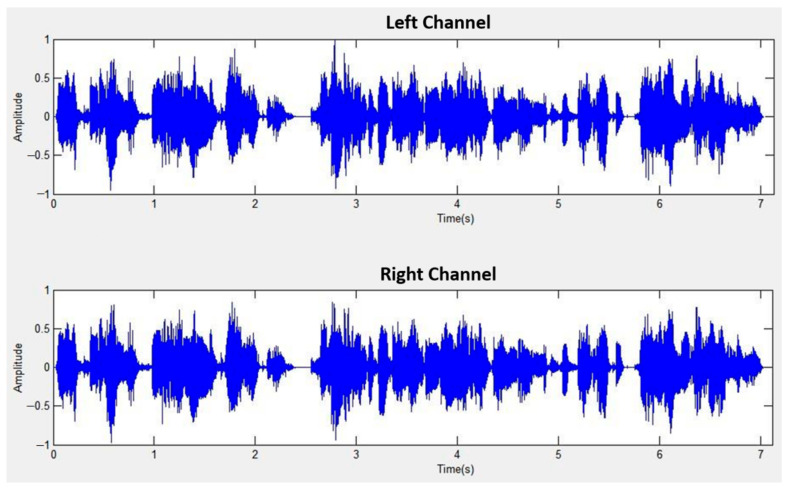
Mixed signal for left and right channels (1 m).

**Figure 15:**
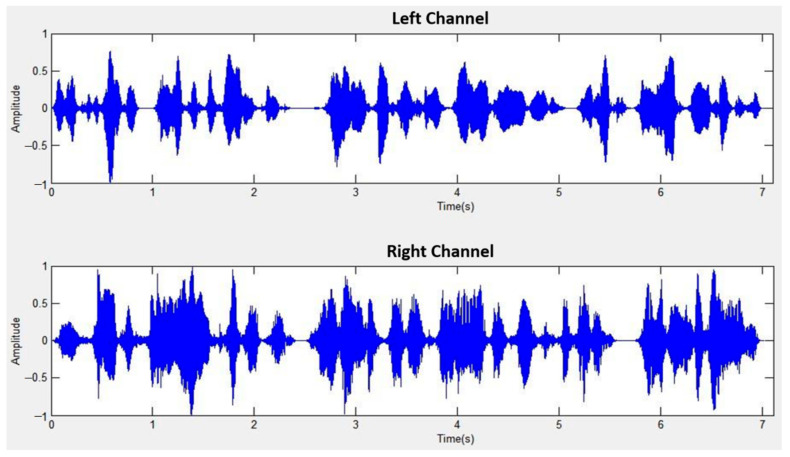
Separated signal after mixed sound source separation (1 m).

**Figure 16:**
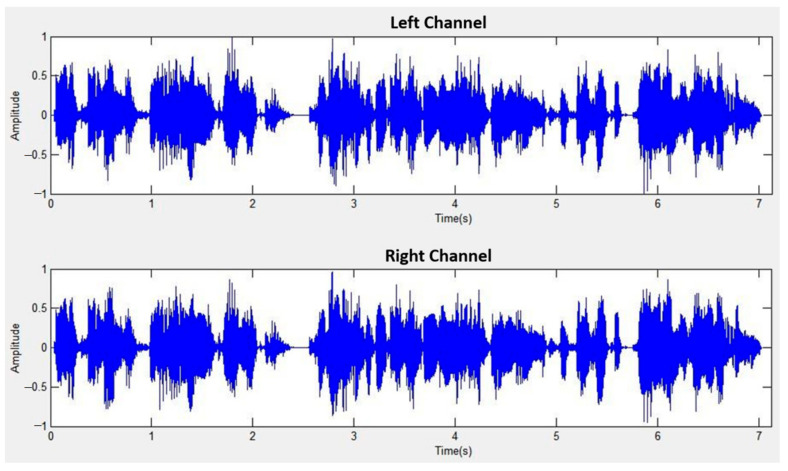
Mixed signal for left and right channels (2 m).

**Figure 17:**
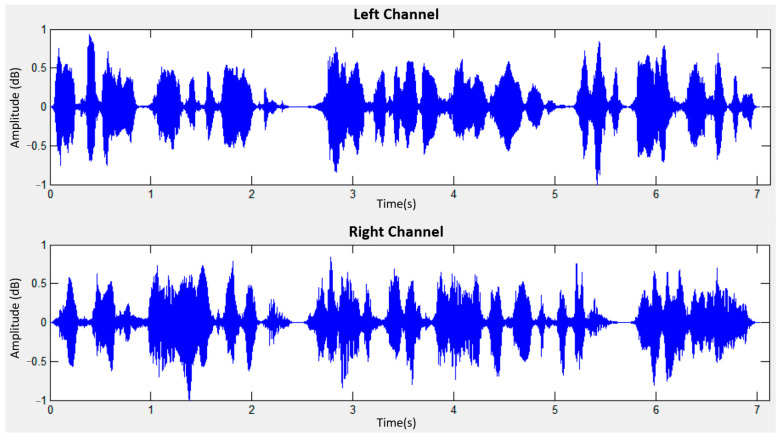
Separated signal after mixed sound source separation (2 m).

The sound source angles (30°, 30°) correspond to 90°, with S1 shifted to the left by 30° and S2 shifted to the right by 30°. Figure 14 displays the mixed signal for left and right channels at a distance of 1 m, while Figure 15 showcases the separated signal after mixed sound source separation at the same distance. Similarly, Figure 16 exhibits the mixed signal for left and right channels at a distance of 2 m, followed by Figure 17 demonstrating the separated signal after mixed sound source separation at the same distance.

For Experimental Setup 2, we utilized the free Speech API provided by Google to evaluate the performance of the algorithm. We tested the algorithm using 20 common commands typically used in a general and simple smart home environment, such as “Open the window,” “Weather forecast,” “Turn off the lights,” “Increase the volume,” “Stock market status,” and so on.

**Figure 18:**
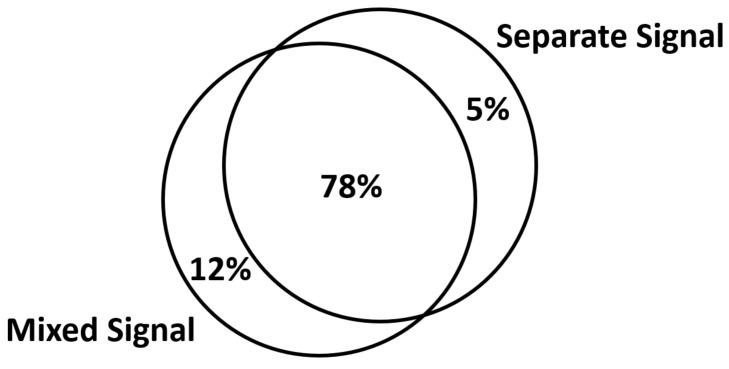
Speech recognition accuracy.

From Table 7, we can observe that the recognition accuracy of the separated signals is lower than that of the mixed signals. However, the recognition accuracy of the separated signals and the mixed signals does not completely overlap. Therefore, by combining the mixed signals and the separated signals (as shown in Figure 18) in a fusion set, we achieved a recognition accuracy of 95%.

## 5. Conclusions and Future Research Directions

On the one hand, this study implemented the orientation detection system on the TI TMS320C6713 DSK development board, and on the other hand, it implemented the hybrid sound source separation system on the Raspberry Pi 2 development board. The experimental results show that the method proposed in this study is better than the hybrid signals, and separated mixed signals enhance the speech recognition capabilities of embedded systems in sensor networks and wearable devices suitable for people’s activity and health monitoring. Firstly, we successfully implemented a direction detection system on the TI TMS320C6713 DSK development board. Secondly, a mixed sound source separation system was implemented on the Raspberry Pi 2 development board.

The received audio signal underwent preprocessing in the direction detection system using voice activity detection (VAD) to identify speech segments. Spectral subtraction was then applied to denoise the noisy audio. Finally, the denoised audio was passed to the DOA (Direction of Arrival) estimator for direction angle detection.

We designed a system with two microphones in the mixed sound source separation algorithm. The received mixed signal was initially transformed from the time domain to the frequency domain using the Fourier transform to exploit its sparsity. Then, feature extraction was performed and the signal was input into the K-Means algorithm for clustering. During the clustering process, a corresponding mask was generated. Here, we used binary masks for separation. The binary-masked mixed signal was then used to reconstruct the source signals in the frequency domain, which was subsequently transformed back to the time domain using the inverse Fourier transform to obtain the separated audio.

In future research, our goal is to further enhance and optimize the algorithm on the embedded board, reduce the computational load of the embedded system, and improve the embedded system’s real-time performance. We also plan to enhance the separation signal quality of the mixed sound source separation algorithm to enhance speech recognition accuracy. In addition, we will try various situation settings and set sound sources at different distances to evaluate the system performance more comprehensively.

## Figures and Tables

**Table 1:** Orientation detection under different environment settings.

Parameters	Value
angle	5°, 15°, 25°, 35°, 45°, 55°, 65°, 75°, 85°, 95°, 105°, 115°, 125°, 135°, 145°, 155°, 165°, 175°
microphone distance	0.22 m
distance between source and microphone center	2 m
sampling frequency	16 kHz

**Table 2:** Test results for direction detection.

Parameters	Value								
actual angle	5°	15°	25°	35°	45°	55°	65°	75°	85°
measured angle	8.53°	8.53°	19.13°	28.54°	41.77°	52.17°	64.12°	75.60°	89.68°
actual angle	95°	105°	115°	125°	135°	145°	155°	165°	175°
measured angle	100.50°	107.69°	118.72°	131.11°	141.17°	152.81°	165.21°	168.54°	180.00°

**Table 3:** Details for mixed sound source separation in Environment 1.

Parameters	Value
angle	30°, 60°, 90°
microphone distance	58 mm
distance between source and microphone center	1 m, 2 m
sampling frequency	8 kHz

**Table 4:** Setup Details for mixed sound source separation in Environment 2.

Parameters	Value
speaker angle	0°, 45°, 90°, 135°, 180°
microphone distance	58 mm
distance between speaker and microphone center	0.2 m
distance between interference source and microphone center	1 m
sampling frequency	8 kHz

**Table 5:** SIR for sound source at 1 m.

Parameters	Value							Average
sound source angle	30°, 30°	30°, 60°	30°, 90°	60°, 30°	60°, 60°	60°, 90°	90°, 90°	
SIR	17.35	17.13	15.64	17.87	17.21	15.76	16.04	16.72

**Table 6:** SIR for sound source at 2 m.

Parameters	Value						Average
sound source angle	30°, 30°	30°, 60°	60°, 30°	60°, 60°	60°, 90°	90°, 90°	
SIR	16.68	16.01	18.76	14.74	15.61	15.74	15.76

**Table 7:** Speech recognition accuracy for mixed sound source separation.

Signal	Speech Recognition Accuracy
mixed signal	90%
separated signal	83%
mixed signal and separated signal	**95%**

## Data Availability

The data presented in this study are openly available.
